# Supplementary material for: Venus’ light slab hinders its development of planetary-scale subduction
Source: Nat Commun. 2022 Dec 10;13:7647. doi: 10.1038/s41467-022-35304-3 (PMC9741584; doi:10.1038/s41467-022-35304-3)
Supplement: Supplementary file 1 — Supplementary information [file 41467_2022_35304_MOESM1_ESM.pdf]

SUPPLEMENTARY INFORMATION

Contents:

Supplementary Note 1

Supplementary Tables 1-4

Supplementary Figures 1-5

Supplementary References

Supplementary Note 1

Equilibrium phase diagram

The equilibrium pressure-temperature (*P*-*T*) phase diagrams show eclogite-facies assemblages (light green area in Supplementary Figs. 1, 2, 3) at P >1.5–2 GPa, T >530 °C. These *P*-*T* areas of eclogite-facies vary among phase diagrams. Vega 2 (Supplementary Fig. 1) has the smallest eclogite stable *P*-*T* area, and Venera 14 (Supplementary Fig. 3) has the largest eclogite-facies *P*-*T* area among the three diagrams. They are, in general, smaller than the eclogite or garnet-pyroxenite areas predicted for Earth's Proterozoic ones (Fig. 1 in Supplementary ref. 1). In the phase diagrams of Vega 2 (Supplementary Fig. 1) and Venera 14 (Supplementary Fig. 3), garnet becomes a stable phase where P >1.1 GPa and T 520-550 °C (higher P and T side of the magenta curve in Supplementary Figs. 1, 3). The stable pressure for garnet in Venera 13 is about 0.5 GPa higher than the other two. Diopside is only stable in a narrow pressure range (0.1–0.2 GPa), up to 550 °C in the Vega 2 diagram (Supplementary Fig. 1). The *P*-*T* area of diopside-bearing assemblage is larger in the Venera13 diagram (Supplementary Fig. 1) and at a higher pressure in the Venera 14 diagram (Supplementary Fig. 3). Omphacite is a stable phase at pressures higher than 2.0–2.2 GPa for the Vega 2 composition and stable at lower temperatures in the Venera 14 diagram (Supplementary Fig. 3) or both lower temperatures and pressures in the Venera 13 diagram (Supplementary Fig. 2).

The amphiboles are actinolite, hornblende and glaucophane in the phase equilibria. Actinolite is an amphibole phase typically stable at low pressure (<1.5 GPa), and low temperature (<500 °C) conditions.

However, actinolite-bearing assemblages are also present in the high-*P*, and low-*T* regimes for Vega 2 (Supplementary Fig. 1) and Venera 14 (Supplementary Fig. 3). Hornblende is a major low *P*/*T* amphibole phase, and glaucophane is stable in high *P*/*T* conditions. The glaucophane stability *P*-*T* area is smaller in the Venera 13 phase diagram (Supplementary Fig. 2), in which glaucophane is replaced by actinolite at high *P*/*T* conditions. As temperature increases, amphiboles dehydrate and are replaced by pyroxenes ± garnet. In high-*P*, high-*T* conditions, glaucophane ± hornblende is replaced by omphacite (Supplementary Figs. 1, 2, 3).

The transition from lawsonite (high-*P*/*T*) to epidote (low-*P*/*T*) takes place at ~2 GPa (at 550 °C) in all three diagrams. Mica minerals include muscovite and biotite with minor paragonite. Biotite is only stable in low-*P* (<1.5 GPa), high-*T* (>500 °C) conditions. Paragonite is only present in the diagrams of Vega 2 and Venera 13 in a small *P*-*T* range (~400–550 °C, ~0.7–1.2 GPa). Other rock-forming minerals in the phase diagrams include zoisite, chlorite, rutile/titanite/ilmenite, kyanite, quartz, and feldspar.

Experimental studies suggest that the hydrous solidus of metabasic system locates at about 700–750 °C (Supplementary Fig. 1). The presence of melt would lower the bulk density but the segregation of melt leaves behind dense restite. The fraction of melt is minor near the hydrous solidus and depends on the water content. While the few indicators of prograde eclogite melting are preserved as leucocratic pockets or glass inclusions[2,3,4], more recorders of eclogite melting are ascribed to decompression melting during exhumation[5,6,7]. The felsic melt cannot escape until its fraction reaches a critical melt porosity of ~30%[8,9]. Partial melting is enhanced by decomposition of hydrous minerals[10], like amphibole[11,12] and phengite[13]. Due to the complexity of activity models and unquantified effects of impurities, these minerals could be stable in a wider *P*-*T* range than the phase diagram predicts (e.g., amphibole in Supplementary Fig. 1), so partial melting is delayed to greater depths. Our phase equilibria modeling predicts largely anhydrous eclogite assemblages at *T* >750 °C, close to the residue phases if melting took place. Thus, a much denser slab is not expected if the resitite remains in the slab crust, and the general estimates are still valid.

The phase relations are insensitive to the variations in bulk Na. In Vega 2 P-X_{Na} diagram (Supplementary Fig. 4a), SiO_2 (quartz) is saturated with lower Na content in the eclogite-facies assemblages. Glaucophane (<2.3 GPa), clinopyroxene (>1.95 GPa), garnet (>1 GPa), and most other minerals' stability boundaries barely vary with the increase of Na content. In Venera 13 diagram (Supplementary Fig. 4b), when the Na concentration increases from 70% to 130%, the actinolite stability area shrinks from >2.5 GPa to 2.0 GPa, and omphacite becomes more stable from >1.1 GPa to <0.5 GPa. The eclogite-facies phase relations are most dependent on the bulk Na content in the phase diagram of Venera14 (Supplementary Fig. 4c). With increasing Na content, garnet is stable at a higher pressure (0.9 GPa at 70% Na to 1.4 GPa at 130%), and omphacite (clinopyroxene) is stable at a lower pressure (1.9 GPa at 70% to 1.0 GPa at 130%). Omphacite (high bulk Na) and diopside (low bulk Na) form a solvus at >2.2 GPa. The stability fields also imply that the mineral modes (vol.%) vary with bulk Na. Although not explicitly depicted in the phase diagram, we can expect decreasing garnet modes and increasing omphacite modes at a specific pressure as the bulk rock become more Na-rich. The effects of these two dense minerals on the bulk density cancel out, so the bulk density remains largely constant as the bulk Na content varies (Fig. 2c).

The bulk compositions of Mg affect several minerals' stability fields (Supplementary Fig. 5). Quartz and zoisite are generally present in Mg-poor assemblages, while the Mg enrichment stabilizes biotite at the expense of muscovite. The stability fields of glaucophane, omphacite, and garnet all shrink to higher pressures to various degrees as the bulk compositions become more Mg-rich (Supplementary Fig. 5), and thus the eclogite-facies assemblages are present at higher pressures. Given that their compositions approach Mg-endmembers that are less dense than Fe-endmembers, the bulk densities of rocks decrease as the bulk compositions are more Mg-rich. In the phase diagram of Venera 14 (Supplementary Fig. 5c), omphacite reacts to form diopside at pressure >2.2 GPa and the Mg concentration higher than 90%. Talc is only stable at >2.3 GPa, with a relatively Mg-rich bulk composition (>90%).

SUPPLEMENTARY TABLE 1. XRF ELEMENTAL ANALYSES OF VENUS' CRUSAL COMPOSITIONS (WT.%).

	SiO_2	TiO_2	Al_2O_3	FeO	MgO	MnO	CaO	K_2O	Na_2O[c]	SO_3	Cl
Vega 2[a]	45.6 ± 3.2	0.2 ± 0.1	16 ± 1.8	7.7 ± 1.1	11.5 ± 3.7	0.14 ± 0.12	7.5 ± 0.7	0.1 ± 0.08	2	4.7 ± 1.5	<0.3
Venera 13[b]	45.1 ± 3.0	1.59 ± 0.45	15.8 ± 3.0	9.3 ± 2.2	11.4 ± 6.2	0.2 ± 0.1	7.1 ± 0.96	4.0 ± 0.63	2.0 ± 0.5	1.62 ± 1.0	<0.3
Venera 14[b]	48.7 ± 3.6	1.25 ± 0.41	17.9 ± 2.6	8.8 ± 1.8	8.1 ± 3.3	0.16 ± 0.08	10.3 ± 1.2	0.2 ± 0.07	2.4 ± 0.4	0.88 ± 0.77	<0.4
Weighted Average	46.2 ± 1.8	0.31 ± 0.09	16.5 ± 1.3	8.2 ± 0.8	9.9 ± 2.3	0.17 ± 0.06	7.9 ± 0.5	0.18 ± 0.05	2.2 ± 0.3[d]	1.7 ± 0.5	

[a] Supplementary ref. 14, Surkov et al. (1986)

[b] Supplementary ref. 15, Surkov et al. (1984)

[c] Estimated in Supplementary refs. 14, 15

[d] The uncertainty of the Na_2O content of Vega 2 is assumed as the average of the other two samples.

SUPPLEMENTARY TABLE 2. BULK-ROCK COMPOSITIONS USED FOR PHASE DIAGRAM CALCULATIONS (MOL. %)

	SiO_2	TiO_2	Al_2O_3	FeO	MgO	CaO	K_2O	Na_2O	O[a]	Sum
Vega 2	51.17	0.17	10.58	7.23	19.24	9.02	0.07	2.18	0.36	100
Venera 13	48.57	1.29	10.03	8.37	18.3	8.19	2.75	2.09	0.42	100
Venera 14	52.1	1.01	11.28	7.87	12.92	11.81	0.14	2.49	0.39	100
Weighted Average	52.01 ± 2.03	0.26 ± 0.08	10.95 ± 0.86	8.11 ± 0.79	16.61 ± 3.86	9.53 ± 0.60	0.13 ± 0.04	2.40 ± 0.33	0.41 ± 0.04	100

[a] Excess oxygen (Fe^{3+}) calculated using $Fe^{3+}/(Fe^{2+}+Fe^{3+})$ = 0.1 (molar ratio), as assumed in Palin and White, 2016.

SUPPLEMENTARY TABLE 3. MINERALS' SOLID-SOLUTION ACTIVITY MODELS AND ABBREVIATIONS.

Minerals	Abbreviation	References
Glaucophane	gl	Supplementary ref. 16, Diener et al. (2007)
Actinolite	act	Supplementary ref. 16, Diener et al. (2007)
Hornblende	hb	Supplementary ref. 16, Diener et al. (2007)
Diopside	di	Supplementary ref. 17, Green et al. (2007)
Omphacite	o	Supplementary ref. 17, Green et al. (2007)
Jadeite	jd	Supplementary ref. 17, Green et al. (2007)
Chlorite	chl	Supplementary ref. 18, Holland et al. (1998)
Garnet	g	Supplementary ref. 19, White et al. (2007)
Epidote	ep	Supplementary ref. 18, Holland & Powell (1998)
Plagioclase	pl	Supplementary ref. 20, Holland & Powell (2003)
Ilminite	ilm	Supplementary ref. 21, White et al. (2000)
Biotite	bi	Supplementary ref. 19, White et al. (2007)
Muscovite	mu	Supplementary ref. 22, Coggon & Holland (2002)
Paragonite	pa	Supplementary ref. 22, Coggon & Holland (2002)
Talc	ta	Supplementary ref. 18, Holland & Powell (1998)

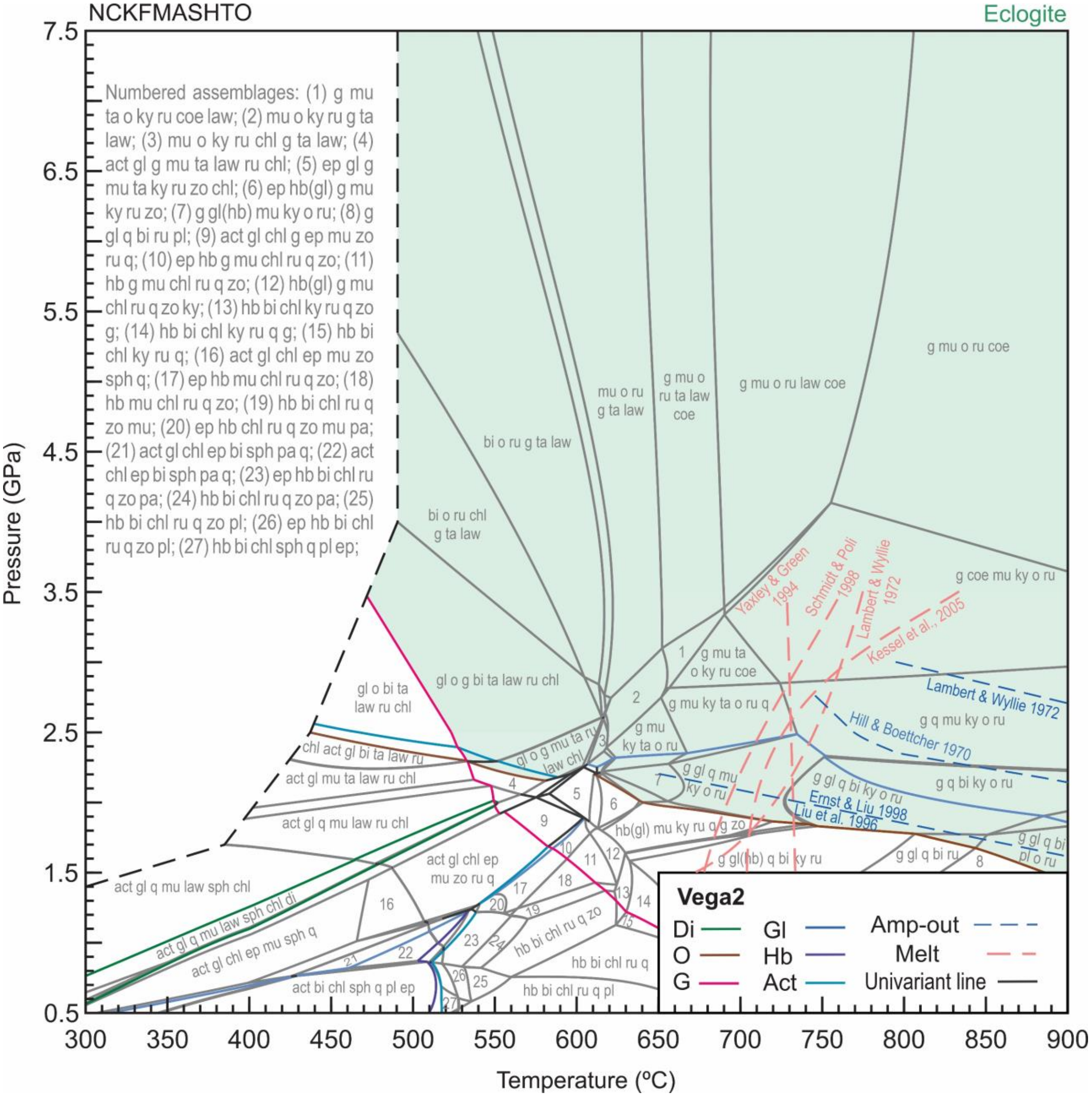

Supplementary Figure 1. Equilibrium *P-T* phase diagram for Vega 2. The phase diagrams are labeled with mineral assemblages and stability boundaries of important phases (garnet, pyroxene, amphiboles). The dashed curves denote phase relations calibrated in experiments (pink: wet solidus of metabasite; blue: stability field of amphibole). The eclogite-facies assemblages are highlighted in light green.

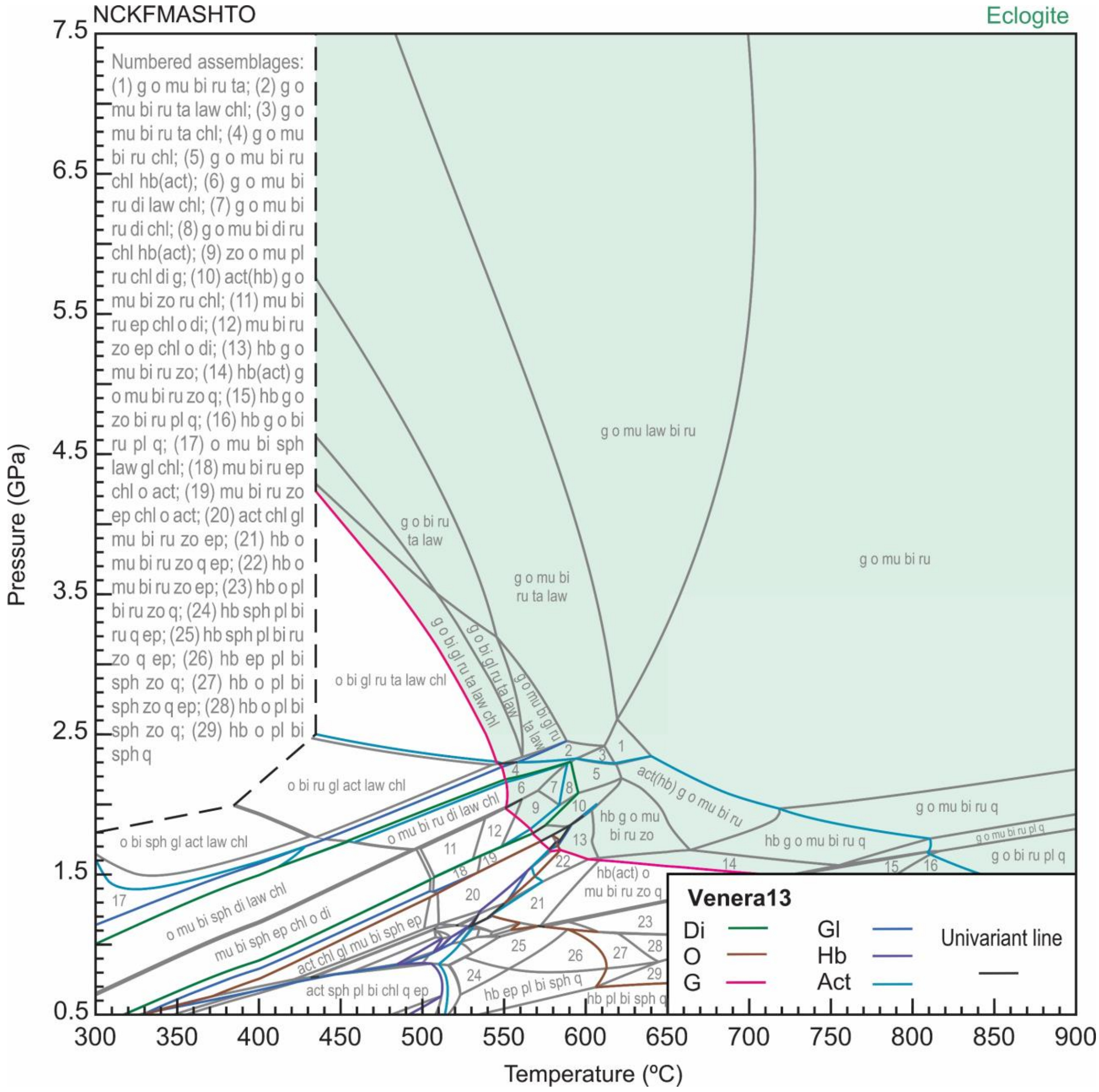

Supplementary Figure 2. Equilibrium *P-T* phase diagram for Venera 13. The phase diagrams are labeled with mineral assemblages and stability boundaries of important phases (garnet, pyroxene, amphiboles). The eclogite-facies assemblages are highlighted in light green.

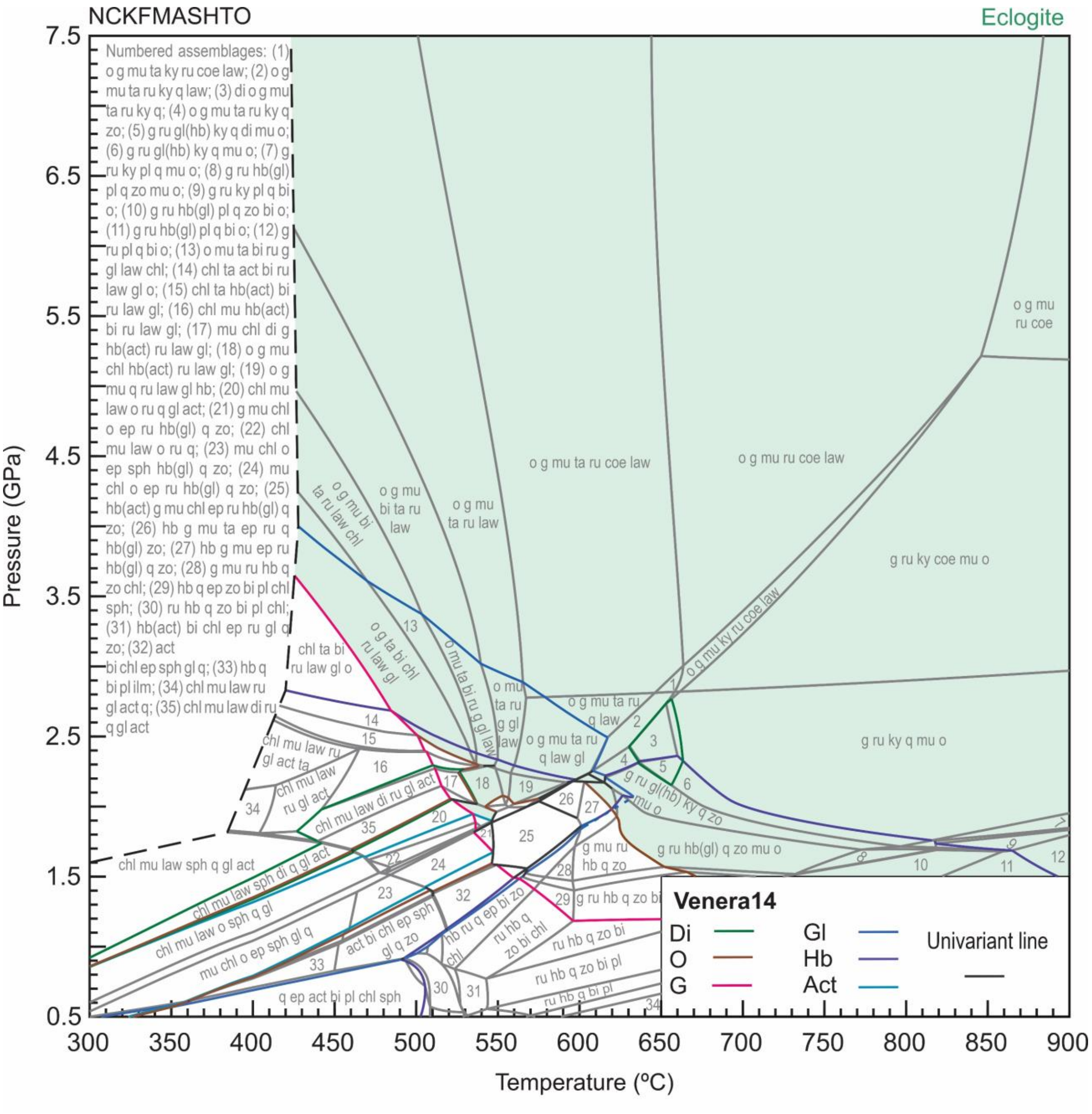

Supplementary Figure 3. Equilibrium *P-T* phase diagram for Venera 14. The phase diagrams are labeled with mineral assemblages and stability boundaries of important phases (garnet, pyroxene, amphiboles). The eclogite-facies assemblages are highlighted in light green.

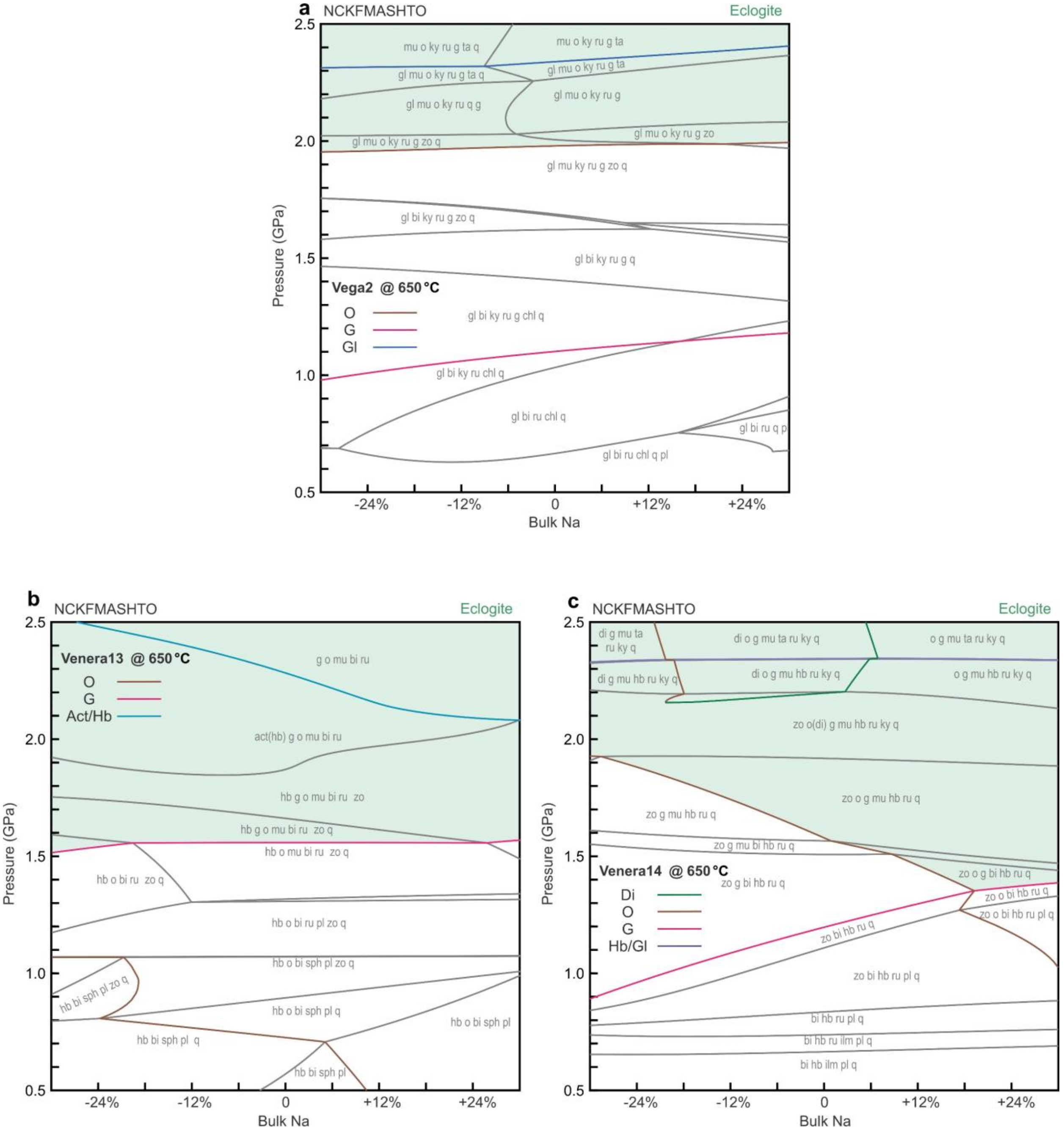

Supplementary Figure 4. *P*-X_{Na} phase diagrams at 650 °C, 0.5–2.5 GPa, with ± 30% bulk Na variations. (a) Vega 2, (b) Venera 13, and (c) Venera 14 compositions ("0" denotes the Na content in Supplementary Table 2). The phase diagrams are labeled with mineral assemblages and stability boundaries of important phases (garnet, pyroxene, amphiboles). The eclogite-facies assemblages are highlighted in light green.

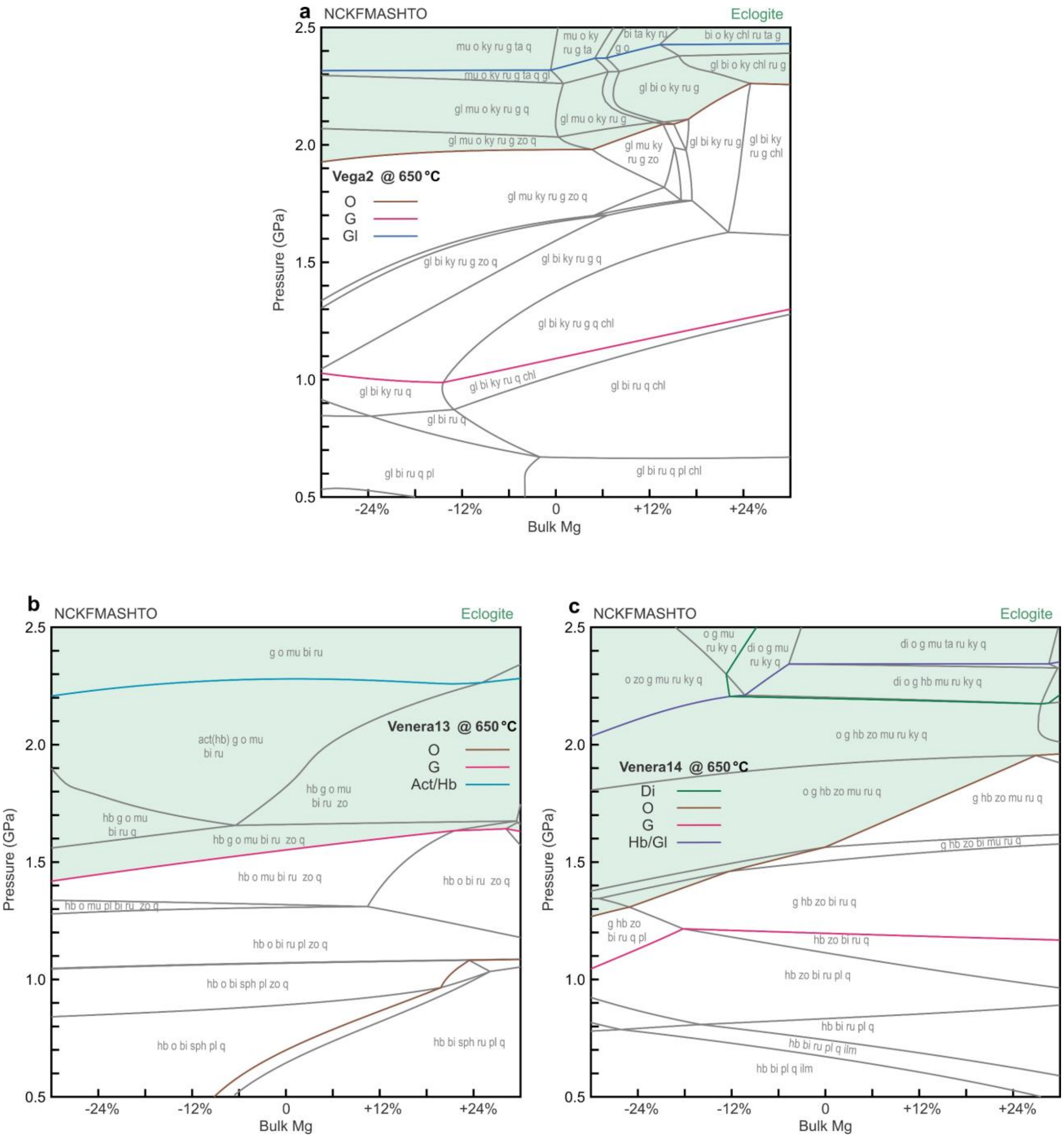

Supplementary Figure 5. *P*-X_{Mg} phase diagram at 650 °C, 0.5–2.5 GPa, with ± 30% bulk Mg variations. (a) Vega 2, (b) Venera 13, and (c) Venera 14 compositions ("0" denotes the Mg content in Supplementary Table 2). The phase diagrams are labeled with mineral assemblages and stability boundaries of important phases (garnet, pyroxene, amphiboles). The eclogite-facies assemblages are highlighted in light green.

Supplementary References

1. Palin, R., White, R. Emergence of blueschists on Earth linked to secular changes in oceanic crust composition. *Nature Geosci* **9,** 60–64 (2016).
2. Okamoto, K., Katayama, I., Maruyama, S. Liou, J.G. Zircon-inclusion mineralogy of a diamond-grade eclogite from the Kokchetav massif, northern Kazakhstan. International Geology Review, **48(10)**, 882-891 (2006).
3. Miyazaki, T., Nakamura, D., Tamura, A., Svojtka, M., Arai, S., Hirajima, T. Evidence for partial melting of eclogite from the Moldanubian Zone of the Bohemian Massif, Czech Republic. *Journal of Mineralogical and Petrological Sciences* **111(6)**, 405-419(2016).
4. Li, Q., Zhang, L., Fu, B., Bader, T., Yu, H. Petrology and zircon U‐Pb dating of well‐preserved eclogites from the Thongmön area in central Himalaya and their tectonic implications. Journal of Metamorphic Geology, **37(2)**, 203-226 (2019).
5. Cao, W., Gilotti, J. A., Massonne, H. J. Partial melting of zoisite eclogite from the Sanddal area, North-East Greenland Caledonides. *European Journal of Mineralogy* **32(4)**, 405-425(2020).
6. Yu, S., Zhang, J., Sun, D., Li, Y., Gong, J., Anatexis of ultrahigh-pressure eclogite during exhumation in the North Qaidam ultrahigh-pressure terrane: Constraints from petrology, zircon U-Pb dating, and geochemistry. *Geol. Soc. Am. Bull.* **127**, 1290–1312(2015).
7. Schorn, S., Hartnady, M. I., Diener, J. F., Clark, C., Harris, C. H_2O-fluxed melting of eclogite during exhumation: an example from the eclogite type-locality, Eastern Alps (Austria). *Lithos* **390**, 106-118(2021).
8. Rapp, R. P. Amphibole‐out phase boundary in partially melted metabasalt, its control over liquid fraction and composition, and source permeability. *Journal of Geophysical Research: Solid Earth* **100(B8)**, 15601-15610(1995).
9. Yu, X., Lee, C.T.A., Critical porosity of melt segregation during crustal melting: Constraints from zonation of peritectic garnets in a dacite volcano. *Earth and Planetary Science Letters* **449**, pp.127-134(2016).
10. Vielzeuf, D., Schmidt, M. W. Melting relations in hydrous systems revisited: application to metapelites, metagreywackes and metabasalts. *Contributions to Mineralogy and Petrology* **141(3)**, 251 (2001).
11. Wyllie, P. J., Wolf, M. B. Amphibolite dehydration-melting: sorting out the solidus. *Geological Society, London, Special Publications* **76(1)**, 405-416 (1993).
12. Cao, W., Gilotti, J. A., Massonne, H. J., Ferrando, S., Foster Jr, C. T. Partial melting due to breakdown of an epidote‐group mineral during exhumation of ultrahigh‐pressure eclogite: An

example from the North‐East Greenland Caledonides. *Journal of Metamorphic Geology* **37(1)**, 15-39 (2019).

13. Schmidt, M. W., Vielzeuf, D., & Auzanneau, E. Melting and dissolution of subducting crust at high pressures: the key role of white mica. *Earth and Planetary Science Letters* **228(1-2)**, 65-84 (2004).
14. Surkov, Y. A., Barsukov, V. L., Moskalyeva, L. P., Kharyukova, V. P., Kemurdzhian, A. L. New data on the composition, structure, and properties of Venus rock obtained by Venera 13 and Venera 14. *J. Geophys. Res.* **89(S02)**, 393–402 (1984).
15. Surkov, Y. A., Moskalyova, L. P., Kharyukova, V. P., Dudin, A. D., Smirnov, G. G., Zaitseva, S. Y. Venus rock composition at the Vega 2 Landing Site. *J. Geophys. Res.* **91(B13)**, 215–218 (1986).
16. Diener, J. F. A., Powell, R., White, R.W. and Holland, T.J.B. A new thermodynamic model for clino- and orthoamphiboles in the system Na_2O–CaO–FeO–MgO–Al_2O_3–SiO_2–H_2O–O. *Journal of Metamorphic Geology* **25**, 631-656 (2007).
17. Green, E., Holland, T. J. B., Powell, R. An order-disorder model for omphacitic pyroxenes in the system jadeite-diopside-hedenbergite-acmite, with applications to eclogitic rocks. *American Mineralogist* **92(7)**, 1181–1189 (2007).
18. Holland, T. J. B., Powell, R. An internally consistent thermodynamic data set for phases of petrological interest. *Journal of Metamorphic Geology* **16** 309-343 (1998).
19. White, R., Powell, R., Holland, T. J. B. Progress relating to calculation of partial melting equilibria for metapelites. *Journal of Metamorphic Geology* **25**, 511-527 (2007).
20. Holland, T., Powell, R. Activity–composition relations for phases in petrological calculations: an asymmetric multicomponent formulation. *Contributions to Mineralogy and Petrology* **145(4)**, 492-501 (2003).
21. White, R., Powell, R., Holland, T. J. B., Worley, B. A. The effect of TiO_2 and Fe_2O_3 on metapelitic assemblages at greenschist and amphibolite facies conditions: mineral equilibria calculations in the system K_2O-FeO-MgO-Al_2O_3-SiO_2-H_2O-TiO_2-Fe_2O_3. *Journal of Metamorphic Geology* **18(5)**, 497-511 (2000).
22. Coggon, R., Holland, T. J. B. Mixing properties of phengitic micas and revised garnet-phengite thermobarometers. *Journal of Metamorphic Geology* **20(7)**, 683-696 (2002).
