# Supplementary material for: Venus’ light slab hinders its development of planetary-scale subduction
Source: Nat Commun. 2022 Dec 10;13:7647. doi: 10.1038/s41467-022-35304-3 (PMC9741584; doi:10.1038/s41467-022-35304-3)
Supplement: Supplementary file 3 — Description of Additional Supplementary files [file 41467_2022_35304_MOESM3_ESM.pdf]

Description of Additional Supplementary Files

Supplementary Movie 1
Description: The evolution of slab temperature as the submerged slab rotates from 0° to 30°. The horizontal white line indicates the crust/mantle boundary, and the white curve in the upper crust portion is the 750 °C isotherm.

Supplementary Movie 2
Description: The evolution of slab density difference from the ambient mantle, for Paleoproterozoic Earth's slab. The horizontal line indicates the crust/mantle boundary, and the hatched area indicates suprasolidus crust.

Supplementary Movie 3
Description: The evolution of slab density difference from the ambient mantle, for Venus (Vega2). The horizontal line indicates the crust/mantle boundary, and the hatched area indicates suprasolidus crust.

Supplementary Movie 4
Description: The evolution of slab density difference from the ambient mantle, for Venus (Venera13). The horizontal line indicates the crust/mantle boundary, and the hatched area indicates suprasolidus crust.

Supplementary Movie 5
Description: The evolution of slab density difference from the ambient mantle, for Venus (Venera14). The horizontal line indicates the crust/mantle boundary, and the hatched area indicates suprasolidus crust.
